# Secondary Squamous Cell Carcinoma of the Tongue Complicated with Bronchiolitis Obliterans as a Manifestation of Graft-versus-Host Disease following Peripheral Blood Stem Cell Transplantation

**DOI:** 10.1155/2019/6015803

**Published:** 2019-11-23

**Authors:** Kengo Hashimoto, Toru Nagao, Shin Koie, Satoru Miyabe, Terumi Saito

**Affiliations:** ^1^Department of Oral and Maxillofacial Surgery, Aichi-Gakuin University School of Dentistry, Nagoya, Japan; ^2^Department of Oral and Maxillofacial Surgery, Okazaki City Hospital, Okazaki, Japan

## Abstract

Peripheral blood stem cell transplantation (PBSCT) has increasingly been used for hematologic cancer therapy, resulting in improved survival rates. However, risks include graft-versus-host disease (GVHD) and secondary solid tumors. Here, we describe a case of tongue squamous cell carcinoma (SCC) complicated by bronchiolitis obliterans (BO) following PBSCT. A 42-year-old man with a history of acute lymphocytic leukemia treated with PBSCT presented with multiple white lesions and erosions on the tongue and buccal mucosa that are compatible with oral chronic GVHD (NIH criteria: score 2). The lesions were presented for 8 years. The patient had a history of BO manifested as GVHD. During follow-up, an exophytic mass was rapidly developed on the left dorsum of the tongue. Biopsy of this lesion confirmed SCC (cT2N0M0). Pulmonary function testing for general anesthesia was almost normal. Hemiglossectomy, supraomohyoid neck dissection, and tongue reconstruction were performed. Thirteen months after surgery, the patient showed neither recurrence of tumor nor progression of oral GVHD. However, the patient died of respiratory failure due to repeated pneumothoraxes and deterioration of BO.

## 1. Introduction

In recent years, peripheral blood stem cell transplantation (PBSCT) has increasingly been used to treat hematologic malignancies, resulting in improved survival rates [1]. However, PSBCT generally has a higher risk of graft-versus-host disease (GVHD) and secondary solid tumor development than bone marrow transplantation [1]. In addition, bronchiolitis obliterans (BO) has been reported in patients undergoing PBSCT [2]. BO is an important respiratory complication which may lead to late morbidity and decreased survival after transplantation. Here, we describe a case of oral squamous cell carcinoma (OSCC) of the tongue complicated by bronchiolitis obliterans (BO) due to GVHD following PBSCT.

## 2. Case Presentation

In March 2012, a 42-year-old man was referred to our department for evaluation of whitish and erosive lesions that involved the tongue, buccal mucosa, palate, and lip. The patient previously received PBSCT for treatment of acute lymphocytic leukemia (ALL) in December 2005; his sister was the related donor, and she matched for HLA antigens. PBSCT was carried out after induction by combined chemotherapy and total body irradiation (TBI). The patient's ALL went into remission without evidence of acute GVHD. Cyclosporine A (100 mg) and prednisolone (10 mg) were utilized for posttransplantation GVHD prophylaxis. However, chronic GVHD (cGVHD) of the skin and eyes were developed 3 months after PBSCT. One month later, airflow obstruction developed, which was considered as evidence of BO. Later, white spots and erosions of the oral mucosa were noted, which persisted without significant change during follow-up until the first visit of our department. In August 2008, he became aware of respiratory discomfort due to pneumothorax of the right lung. The pneumothorax was successfully treated with a thoracic cavity drainage.

**Figure 1:**
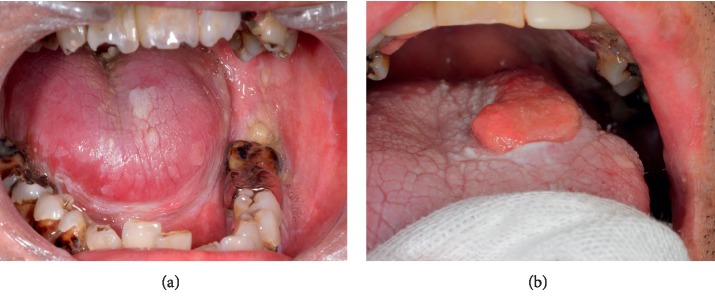
(a) A 42-year-old man presenting with lichenoid changes and atrophic mucosa located on the buccal mucosa, tongue, and lip, compatible with oral chronic graft-versus-host disease. Multiple dental caries is present due to poor oral hygiene associated with xerostomia. (b) An exophytic mass (20 × 15 mm) with induration on the left dorsum of the tongue base with a background of lichenoid mucositis.

Intraoral examination revealed lichenoid changes and atrophic mucosa located on the buccal mucosa, tongue, and lip, compatible with oral cGVHD (Figure 1(a)). The patient was received plaque control instruction to improve poor oral hygiene associated with severe xerostomia. A lip biopsy for confirmation of oral cGVHD was performed on June 7, 2012. The biopsy specimen demonstrated a lichenoid infiltration compatible with cGVHD (NIH criteria: score 2).

**Figure 2:**
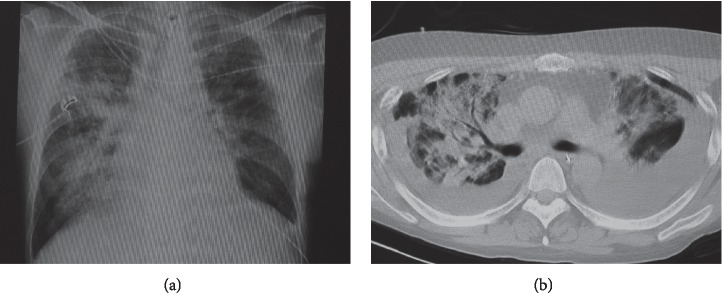
(a) Plain chest radiography showing bilateral infiltrations on the 7^th^ day after surgery. (b) Chest computed tomography imaging showing a significant ground-glass pattern, compatible with acute respiratory distress syndrome.

In January 2013, 8 years after PBSCT, a raised mucosal lesion appeared on the left dorsal surface of the tongue base, with a background of lichenoid mucositis. This lesion decreased in size by 50% over the next month. However, the lesion then enlarged over the following 3 months and appeared as an exophytic mass (20 × 15 mm) (Figure 1(b)). The lesion was then biopsied; histopathologic examination showed well-differentiated OSCC (T2N0M0: stage II). After preoperative examination, we determined that the patient had adequate cardiorespiratory function to undergo an invasive operation under general anesthesia. In July 2013, hemiglossectomy and left supraomohyoid neck dissection and reconstruction with a rectus abdominis musculocutaneous flap were performed after cessation of immunosuppressants. Histopathologically, the excised specimen revealed a well-keratinized OSCC with negative tumor margins; regional lymph nodes were negative for metastasis. On the 7^th^ day after the operation, the patient's respiratory status was suddenly deteriorated. Bilateral infiltrations were noted on plain chest radiography (Figure 2(a)), and significant ground-glass abnormalities were seen on a chest computed tomography (Figure 2(b)), indicating acute respiratory distress syndrome (ARDS). Subsequently, mechanical ventilation, intravenous antibiotics and sivelestat sodium, and control of body fluids were administered. The ARDS improved over two weeks. Adjuvant therapy for OSCC was not considered, given the patient's status. Although the surgical site remained clinically stable, approximately 2 months later, recurrent pneumothorax developed on the right. In October 2013, the patient was discharged after the pneumothorax improved. Thirteen months after surgery, the patient showed neither recurrence of tumor nor progression of oral GVHD. However, the patient died of respiratory failure due to repeated pneumothorax and deterioration of BO.

## 3. Discussion

The incidence of oral cancer as a secondary malignancy in patients with previous hematopoietic stem cell transplantation (HSCT) is 4 to 7 times that of the general population [3]. The risk for developing a malignant neoplasm after PBSCT is 3.5% at 10 years and 11.5% at 15 years; the overall incidence of malignant tumors is approximately 5 folds greater after adjusting for age and sex [3]. SCC of the skin and mouth accounts for one-third of all solid second malignancies. OSCC represents 50% of these cases [4]. Kruse and Gratz [3] reported that the most OSCC appear 5 to 9 years after HSCT. Several risk factors for the development of solid tumors after HSCT have been previously discussed, including TBI, chemotherapy, male gender, viral infection, young age, cGVHD, and immunosuppressive therapy [3–5]. Of these, cGVHD has been considered to be a significant risk factor for the development of any solid tumor, particularly oral and esophageal cancer. Oral lesions are common in patients with cGVHD and occur in 45%–83% of patients within 3 years after HSCT [6]. Any oral site may be involved, with the buccal mucosa and tongue among most commonly affected [6]. Moreover, Curtis et al. [1] reported that cGVHD may be more intense and more frequent in general after PBSCT, which may alter the risk for subsequent SCC.

Oral manifestations of cGVHD can be characterized as mucosal or salivary sclerotic in nature and resemble several autoimmune conditions, including Sjogren's syndrome, oral lichen planus, and scleroderma in both clinical features and histological appearance [7]. Oral cGVHD is characterized by lesions clinically similarly to lichen planus, a relatively common disorder of the oral cavity with a malignant potential. Long-term immunologic damage to the mucosa by T cells has been implicated in predisposing this tissue to malignant transformation. Imanguli et al. [6] noted that multiple factors must be considered when treating a patient with oral GVHD. Even though oral GVHD may have serious health consequences, GVHD of the liver or lungs is life-threatening. Therefore, therapeutic decisions must include consideration of the patient's medical regimen for nonoral GVHD conditions; cooperation between treating physicians and dentists is required. Systemic immunosuppressive therapy is generally used for treatment of cGVHD. Its primary limitation is the increased risk for opportunistic infections, a leading cause of mortality in HSCT patients [6]. Moreover, chronic inflammation induced by systemic immunosuppressive therapy has also been identified as a potential risk factor for secondary oral cancer [4]. The interactions between chronic inflammation and therapy-induced immunosuppression are not fully understood, but immunosuppression in the context of chronic inflammation, as in cGVHD, may interfere with tissue repair, thereby promoting the risk for tumor development [8]. In our case, long-term immunosuppression and chronic inflammation associated with cGVHD and poor oral hygiene were potential risk factors, which are considered to play a pivotal role in carcinogenesis after PBSCT. The differential diagnosis for OSCC in patients with cGVHD includes hyperplastic candidiasis, papilloma virus-induced verrucae, herpetic mucositis, and benign tongue nodule.

The occurrence of tongue OSCC located on the dorsum is rare, even in the Japanese population, accounting for only 3%–5% of cases [9]. OSCC of the dorsum of the tongue is characterized by the existence of predisposing conditions such as long-standing lichen planus, median rhomboid glossitis, Fanconi's anemia, and cGVHD of the oral mucosa [9]. Moreover, OSCC after HSCT has been reported to be more aggressive than in non-HSCT patients [4]. In our case, the reconstructive surgery was required due to the aggressiveness of the tumor and its location on the dorsum and nearby tongue base, which can greatly influence the ingestion and swallowing function. As a result, this invasive treatment was considered to be a possible cause of the patient's postoperative severe pulmonary complications.

Pulmonary complications are a major cause of morbidity and mortality in transplant recipients, occurring in 40%–60% of patients [2]. Though relatively uncommon, BO may result in late morbidity and decreased survival after HSCT [2, 9]. Previously described risk factors are existence of GVHD, PBSCT, a long interval from diagnosis to transplant, female donor to male recipient, and a prior episode of interstitial pneumonitis [2, 9]. To date, there are no definitive criteria or guidelines regarding appropriateness of general anesthesia for BO patients. Surgical decision-making should consider pulmonary function and respiratory symptoms. We determined that the patient's pulmonary function could enough tolerate an operation under general anesthesia. However, postoperative management required strict control of respiratory function and body fluids due to the development of ARDS. Invasive surgery and the load of general anesthesia may have triggered the development of repeated pneumothoraxes and deterioration of BO. The mechanism of pneumothorax associated with BO is thought to occur from the rupture of subpleural blebs [10].

In conclusion, transplant recipients should be followed closely to detect early OSCC or precursor lesions. However, the difficulty in discriminating malignant changes from oral GVHD lesions must be recognized because of atypical clinical tumor features and the wide distribution of oral GVHD lesions throughout the oral cavity. Moreover, Elad et al. [11] noted an increased risk for recurrence of OSCC in patients after HSCT. From comparison of their cases, our case might be at high risk for recurrence. The surveillance should be frequent in patients with OSCC post-HSCT, and repeated biopsies should be considered whenever recurrence is suspected.
